# Postharvest light-induced flavonoids accumulation in mango (*Mangifera indica* L.) peel is associated with the up-regulation of flavonoids-related and light signal pathway genes

**DOI:** 10.3389/fpls.2023.1136281

**Published:** 2023-03-13

**Authors:** Wencan Zhu, Hongxia Wu, Chengkun Yang, Bin Shi, Bin Zheng, Xiaowei Ma, Kaibing Zhou, Minjie Qian

**Affiliations:** ^1^ Sanya Nanfan Research Institute & Key Laboratory of Quality Regulation of Tropical Horticultural Crop in Hainan Province, School of Horticulture, Hainan University, Haikou, China; ^2^ Key Laboratory of Tropical Fruit Biology, Ministry of Agriculture and Rural Affairs, South Subtropical Crops Research Institute, Chinese Academy of Tropical Agricultural Sciences, Zhanjiang, China

**Keywords:** mango, flavonoids, light treatment, metabolites profile, gene expression

## Abstract

**Introduction:**

Flavonoids are important secondary metabolites in plants and light is a crucial environmental factor regulating flavonoids biosynthesis. However, effect of light on the different flavonoids compositions accumulation in mango and the relevant molecular mechanism still need to be clarified.

**Methods:**

In this study, green-mature fruits of red mango cultivar ‘Zill’ were subjected to postharvest light treatment, and fruit peel color, total soluble solids content, total organic acid, and firmness of flesh were measured. The flavonoids metabolites profile, and the expression of flavonoids-related genes and light signal pathway genes were also analyzed.

**Results:**

Results showed that light treatment promoted the red coloration of fruit peel and increased the total soluble solids content and firmness of flesh. The concentration of flavonols, proanthocyanidins and anthocyanins, and expression of key flavonoids biosynthetic genes including *MiF3H*, *MiFLS*, *MiLAR*, *MiANS*, *MiUFGT1*, and *MiUFGT3* were significantly induced by light. The MYBs regulating flavonols and proanthocyanidins, i.e. MiMYB22 and MiMYB12, as well as the key light signal pathway transcription factors (TFs) MiHY5 and MiHYH, were identified in mango. The transcription of *MiMYB1*, *MiMYB12*, *MiMYB22*, *MiHY5* and *MiHYH* was up-regulated by light.

**Discussion:**

Our results provide a postharvest technology to improve mango fruit appearance quality, and are helpful to reveal the molecular mechanism of light-induced flavonoids biosynthesis in mango.

## Introduction

Flavonoids are important secondary metabolites in plants which determine fruit quality due to their essential contribution to fruit color, antioxidation capacity and nutritive value. Flavonoids consist of various classes including flavonols, proanthocyanidins (PAs), and anthocyanins, which are the three main subgroups (Williams and Grayer, 2004). Flavonoids are synthesized *via* phenylpropanoid and flavonoid pathway (Winkel-Shirley, 2001). The early flavonoids biosynthetic genes (EBGs) include Phenylalanine ammonia-lyase (*PAL*), chalcone synthase (*CHS*), chalcone isomerase (*CHI*), flavanone 3-hydroxylase (*F3H*), and flavonoid 3’-hydroxylase (*F3’H*). The late flavonoids biosynthetic genes (LBGs) involve dihydroflavonol reductase (*DFR*), flavonol synthase (*FLS*), anthocyanidin synthase (*ANS*), leucoanthocyanidin reductase (*LAR*), anthocyanidin reductase (*ANR*), and UDP-glucose: flavonoid 3-*O*-glucosyltransferase (*UFGT*). Among them, FLS, UFGT, and LAR and ANR catalyze the last step of flavonols, anthocyanins, and proanthocyanidins biosynthesis, respectively.

The expression of flavonoids biosynthetic genes is regulated by MYB-bHLH-WD40 (MBW) complex, with the crucial contribution of R2R3-MYB transcription factor (TF) (Broun, 2005). In Arabidopsis, total 125 R2R3-MYB TFs are divided into 25 subgroups, and the 5^th^, 6^th^ and 7^th^ subgroups participate in the biosynthesis of proanthocyanidins, anthocyanins, and flavonols, with the representative MYB123/TT2, MYB75/PAP1, and MYB12/PEG1, respectively (Stracke et al., 2001; Dubos et al., 2010). The MYBs controlling flavonoids biosynthesis in fruits have also been widely reported. In strawberry, FaMYB9/FaMYB11, the homologues of AtTT2, were found to form the complex with FabHLH3 and FaTTG1 to control proanthocyanidins biosynthesis in strawberry fruits (Schaart et al., 2013). Proanthocyanidins and flavonols accumulation in red-fleshed apple are regulated by MYB12 and MYB22, respectively (Wang et al., 2017). PpMYB17 could activate the expression of *PpCHS*, *PpCHI*, *PpF3H*, and *PpFLS* to positively regulate flavonols biosynthesis in pear (Premathilake et al., 2020). With the great contribution to the red coloration in fruits, MYBs regulating anthocyanins biosynthesis have been identified in diverse fruit species, including MdMYB1/A/10 in apple (Takos et al., 2006; Ban et al., 2007; Espley et al., 2007), PyMYB10 and PyMYB114 in pear (Feng et al., 2010; Yao et al., 2017), VvMYBA1 in grape (Kobayashi et al., 2004), and CsRuby in citrus (Butelli et al., 2012; Huang et al., 2018; Huang et al., 2019). In mango, the anthocyanins biosynthesis is regulated by MiMYB1 (Kanzaki et al., 2020; Shi et al., 2021), while more MYBs controlling other flavonoids compositions biosynthesis need to be discovered.

Flavonoids biosynthesis in fruits is affected by environmental factors such as light. Postharvest light treatment is widely used to induce flavonoids accumulation in numerous fruit species including apple (Peng et al., 2012), pear (Qian et al., 2013; Sun et al., 2014), peach (Santin et al., 2019), nectarine (Scattino et al., 2014), grape (Sheng et al., 2018), tomato (Liu et al., 2011), and blueberry (Yang et al., 2019). CONSTITUTIVELY PHOTOMORPHOGENIC 1 (COP1), ELONGATED HYPOCOTYL 5 (HY5), and HY5-HOMOLOG (HYH) are the key proteins regulating photomorphogenesis in plants such as flavonoids accumulation (Podolec et al., 2021). COP1 is an ubiquitin E3 ligase, which negatively regulate light-induced flavonoids biosynthesis by degrading flavonoids-related TFs including MYB (Li et al., 2012), and bHLH (Tao et al., 2020). HY5 and HYH are light-responsive TFs, which positively regulate flavonoids accumulation in plants by activating the expression of flavonoids-related genes including *CHS*, *ANS*, *FLS*, and *MYB*, through the binding to the G-box or ACE-box in the promoter region of target genes (Holm et al., 2002; Loyola et al., 2016; Henry-Kirk et al., 2018; Tao et al., 2018).

Mango (*Mangifera indica* L.) is one the most popular tropical fruit species due to its unique aroma, flavor, and enriched nutrition. Following banana, grape, apple and orange, mango is the fifth most produced fruit crop worldwide (http://www.fao.org/faostat/). The effect of light on flavonoids accumulation in mango has been reported. Preharvest bagging treatment inhibited the accumulation of flavonols and anthocyanins but promoted the accumulation of proanthocyanidins in mango (Shi et al., 2021). Postharvest light treatment could increase the total phenols, total flavonoids, and anthocyanins content in the fruit skin of mango (González-Aguilar et al., 2007; Cao et al., 2016; Ni et al., 2022; Shi et al., 2022). However, the effect of postharvest light treatment on the accumulation of different flavonoids components, i.e. flavonols, proanthocyanidins and anthocyanins is still unknown.

In this study, green mature bagged ‘Zill’ mango fruits were subjected to postharvest light treatment, and fruit peel was sampled at 0, 6, 24, 72, 144, and 240 hours of exposure. Fruit quality indexes including firmness, fruit color, total soluble solids content, total organic acid content, and solidity-acid ratio were measured. Metabolomic profiling was established to analyze the concentration of flavonols, proanthocyanidins and anthocyanins in the fruit peel during treatment. The expression of flavonoids biosynthetic and regulatory genes (especially different *MYBs*), as well as the key light signal pathway genes including *COP1*, *HY5* and *HYH*, was also measured. This study will enrich our knowledge regarding the mechanism of light-induced flavonoids biosynthesis in mango.

## Materials and methods

### Plant materials and treatments

Mango (*Mangifera indica* cv. Zill) fruits were obtained from South Subtropical Crops Research Institute (SSCRI) in Zhanjiang, China. Fruitlets were bagged with double layers yellow-black paper bags (Qingdao Kobayashi Co., Ltd., Qingdao, China) at 20 days after full bloom to block out all the light. Green mature fruits (130 days after full bloom) were harvested with bags, transported to the lab, and debagged for postharvest light treatment in plant growth chambers (Conviron, Adaptis A 1000, Winnipeg, Canada). 180 unblemished fruits with uniformed size were divided into two groups, with half fruits subjected to mimic sunlight treatment (mixture of 4.5 μW•cm^-2^ UV-B and 16 W•m^−2^ white light) and the rest retained in darkness as control. The relative humidity and temperature were 80% and 17°C, respectively. All the conditions for treatment were according to our previous patent (Qian et al., 2022). 30 fruits were regarded as one biological replicate. Fruit peel of 5 fruits per replicate was collected at 0, 6, 24, 72, 144, and 240 hours of light exposure for metabolomic and gene expression analyses. For fruit peel sampling, the exposed side was sampled by a peeler for light-treated fruit, and the up-side fruit peel was sampled for control fruit. Fruit peel was sampled as thin as possible to ensure the minimum collection of flesh.

### Fruit quality measurement

Firmness was measured by a TA touch texture profile analyzer (Bosin Tech, Shanghai, China). After removing the peel, a 2 mm diameter probe was inserted to the equatorial part of the flesh at a 90 °C angle and depth of 5 mm. The analyzer parameters for operation were set as follows: 2 mm s^-1^ for pre-test speed; 4 mm s^-1^ for test speed; 3 mm s^-1^ post-test speed, and 2 s for intermediate interval. Fruit color indexes in the equatorial part of fruit (L*, a*, and b* values) were measured by a portable colorimeter (LS170, Shenzhen Linshang Technology Co.,Ltd., Shenzhen, China) according to the instruction of the manufacturer. Total soluble solids content, total organic acid, and solidity-acid ratio were measured by a brix-acidity meter (PAL-BX/ACID15, ATAGO, Tokyo, Japan) according to the user manual. Analysis was performed in three biological replicates.

### Metabolomic profiling of flavonoids

The details for metabolomic profiling of flavonoids were described in the previous study (Shi et al., 2021), which was conducted by Metware Biotechnology Co. Ltd. (Wuhan, China). In brief, fruit peel was successively freeze-dried, ground, and added to the extraction solution (50% methanol containing 0.1% HCl), and the supernatant was used for high-performance liquid chromatography with tandem mass spectrometry (HPLC−MS/MS) analysis. The identification of flavonoids compounds was based on the Metware Database (MWDB). The quantification of flavonoids was according to the area of the chromatographic peak, and the concentration was calculated by using the linear equation of corresponding standard. Analysis was performed in three biological replicates.

### RNA extraction, cDNA synthesis, and Q-PCR analysis from mango peel

Total RNA was extracted using an RNA prep pure plant kit (Tiangen, DP441, Beijing, China). First-strand cDNA was synthesized from 1 µg of total RNA using the HiScript IIQ RT SuperMix (Vazyme, R223-01, Nanjing, China). The Q-PCR primers were designed by Primer 3 (https://bioinfo.ut.ee/primer3-0.4.0/) and synthesized by Sangon Biotech Co. Ltd, Shanghai, China (**Table S1**). The Q-PCR reactions (15µL) were performed on a real-time PCR machine (qTOWER3G, Jena, Germany) containing 7.5µL SYBR premix ExTaqTMII (Takara, Japan), 5.5µL of cDNA (20 times diluted) and 1µL of both forward and reverse primers (10µM). Relative expression of mRNA was calculated by the cycle threshold (Ct) 2^-ΔΔCt^ method, with the normalization by the mango *actin* gene.

### Identification, multiple sequence alignment and phylogenetic tree construction of MYBs, HY5, and HYH

Apple MdMYB22 (AAZ20438.1), MdMYB12 (XP_008337875.1), MdHY5 (NP_001280752.1), and MdHYH (XP_008369576.1) sequences were used to search for the mango homologs by blasting to mango genome database using TBtools (Chen et al., 2020; Wang et al., 2020a). The amino acid sequences of R2R3 domain of flavonoids-related MYBs, and the whole amino acid sequences of HY5 and HYH from mango and other plant species were used for multiple sequence alignment by Jalview (Waterhouse et al., 2009). Full-length flavonoids-related MYBs, HY5, HYH protein sequences were used for phylogenetic tree construction by MEGA X with the neighbor-joining (NJ) method.

### Statistical analysis

Data of fruit quality indexes were subjected to a one-way Analysis of Variance (ANOVA) using SPSS 27.0 (SPSS, Chicago, IL, USA), and mean values were separated by Tukey’s multiple range test. Probability values of <0.05 were considered statistically significant. Metabolic and gene expression data were subjected to a Student’s *t*-test using SPSS 27.0 to analyze the statistic difference between control and treatment. Probability values of <0.05 and <0.01 were considered statistically significant and highly statistically significant, marked with one asterisk (*) and two asterisks (**), respectively. Correlation analysis was conducted by ChiPlot (https://www.chiplot.online/).

## Results

### Effect of postharvest light exposure on the fruit quality of mango

**Figure 1:**
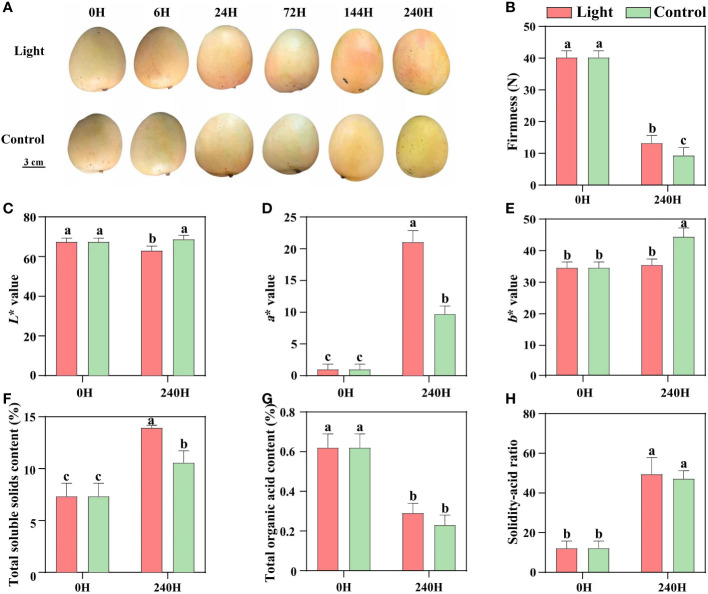
Effect of light on the appearance quality and internal quality of ‘Zill’ mango. **(A)** Representative images of light-treated and control (darkness) ‘Zill’ mango fruits. **(B)** Effect of light treatment on flesh firmness. **(C–E)** Effect of light treatment on fruit color indexes L*, a* and b*. **(F–H)** Effect of light treatment on the total soluble solids content, total organic acid content and solidity-acid ratio in mango flesh. Each value represents the mean ± standard deviation of three biological replicates. Values without the same letter are significantly different, *p* < 0.05 according to Tukey tests.

During postharvest light exposure, no red coloration was detected in the control fruits which were kept in darkness, while the light-treated fruits started turning red at 6 days of exposure, and the red coloration was enhanced at 10 days (**Figure 1A**). After 10 days of treatment, the firmness of both light-treated and control fruits was decreased, and control fruits showed significant lower firmness than light-treated fruits (**Figure 1B**). Red coloration decreased the lightness (L* value), and yellowness (b* value) of light-treated fruits, but tremendously increased the fruit redness (a* value) (**Figures 1C–E**). Light treatment increased the total soluble solids content, but had no effect on the total organic acid content and solidity-acid ratio (**Figures 1F–H**).

### Effect of postharvest light exposure on the concentration of different flavonoids compositions in mango fruit peel

**Figure 2:**
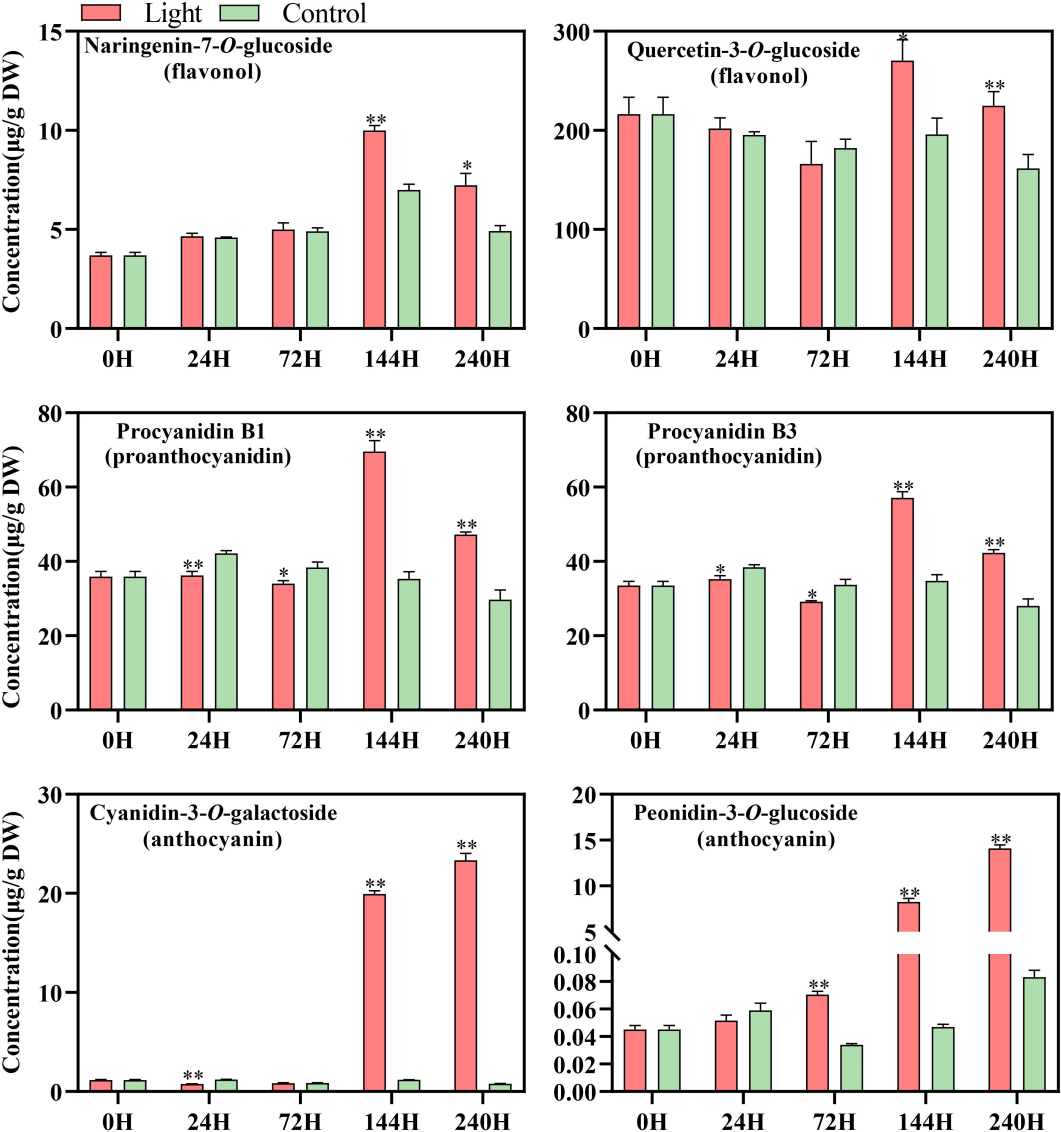
Concentration of flavonoid compounds detected in the fruit skin of light-treated and control (darkness) ‘Zill’ mango fruits during different postharvest light treatment stages. Each value represents the mean ± standard deviation of three biological replicates. * indicates significant difference (*p*-value < 0.05), and ** indicates very significant difference (*p*-value < 0.01) between control and treatment, as determined by Student’s *t*-test.

A total of 108 standards include 9 flavonols, 6 proanthocyanidins, and 93 anthocyanins, while most substance could not be detected or exists at a very low level in our sample (**Table S2**). According to the results of metabolites profile, naringenin-7-*O*-glucoside and quercetin-3-*O*-glucoside, procyanidin B1 and procyanidin B3, and cyanidin-3-*O*-galactoside and peonidin-3-*O*-glucoside were the main flavonols, proanthocyanidins, and anthocyanins components in ‘Zill’ mango fruit peel, respectively (**Figure 2**). The increase of flavonoids concentration was detected by light treatment, while accumulation pattern differed among components (**Figure 2**). The concentration of flavonols and proanthocyanidins in light-treated sample peaked at day 6, and then decreased at day 10 but still with a relatively higher concentration (**Figure 2**). For anthocyanins, significant accumulation of Cyanidin-3-*O*-galactoside, and Peonidin-3-*O*-glucoside were detected at day 6 and day 3, respectively, continued and peaked at day 10 (**Figure 2**). Control samples showed very low content of anthocyanins, but accumulated certain amount of flavonols and proanthocyanidins (**Figure 2**). Concentration of most flavonoids compositions was relatively stable in control samples during the whole treatment (**Figure 2**).

### Effect of postharvest light exposure on the structural genes expression of flavonoids biosynthesis in mango fruit peel

**Figure 3:**
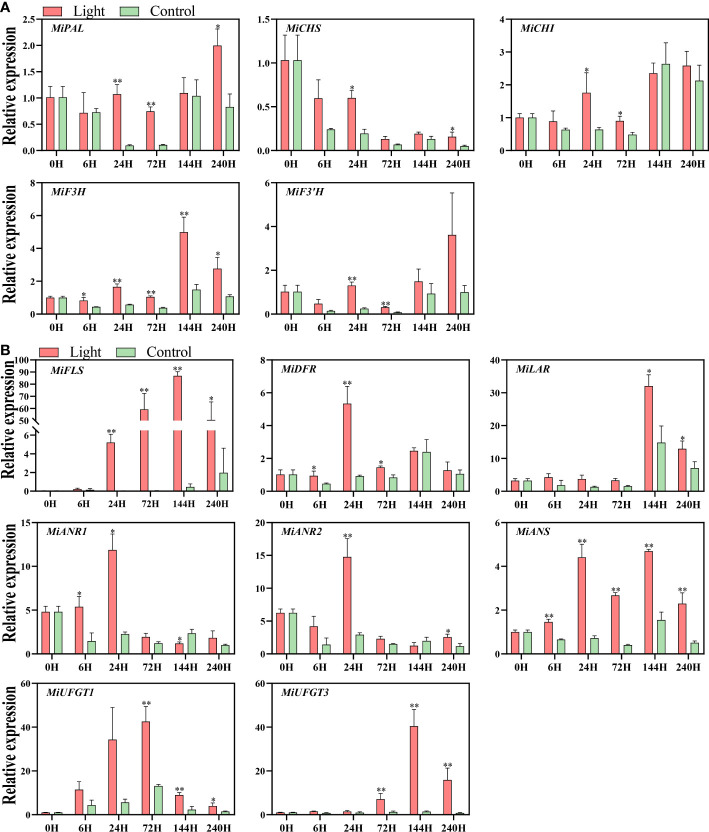
Effect of light treatment on the expression of early flavonoids biosynthetic genes **(A)** and late flavonoids biosynthetic genes **(B)** in ‘Zill’ mango peel. Each value represents the mean ± standard deviation of three biological replicates. * indicates significant difference (*p*-value < 0.05), and ** indicates very significant difference (*p*-value < 0.01) between control and treatment, as determined by Student’s *t*-test.

Light up-regulated the expression of all the structural genes of flavonoids biosynthesis, while the expression pattern differed among genes (**Figures 3A, B**).

For EBGs, the expression of *MiPAL*, *MiCHI*, and *MiF3’H* was increased at day 1 and day 3 by light treatment, and *MiCHS* expression was induced at day 1 and day10 (**Figure 3A**). The expression of *MiF3H* was increased at 6 hours of light treatment, and stayed at a higher level in the light-treated fruits during the whole experiment, with the expression peak at day 6 (**Figure 3A**).

For LBGs, the increase of *MiDFR* expression was detected at 6 hours, 1 day, and 3 days of exposure, and up-regulation of *MiANS* started from 6 hours of treatment and lasted during the whole treatment (**Figure 3B**). As a flavonols specific gene, *MiFLS* expression of light-treated sample was tremendously induced at day 1, peaked almost at day 6, and kept at a high expression level during the treatment (**Figure 3B**). For proanthocyanidins specific genes, *MiLAR* transcription in light-treated fruits was up-regulated at day 6 and day 10, with the peak at day 6, which is highly correlated to the proanthocyanidins concentration (**Figure 3B**). The expression of *MiANR1* responded at the early stages of light treatment, i.e. 6 hours and 1 day, while *MiANR2* expression was significantly up-regulated in light-treated fruits at day1 and day 10 (**Figure 3B**). For anthocyanins specific genes, the increased expression of both *MiUFGT1* and *MiUFGT3* was detected at day 3, day 6, and day10, and the expression peak of *MiUFGT1* and *MiUFGT3* occurred at day 3 and day 6, respectively (**Figure 3B**).

### Identification of flavonoids-related MYBs and expression of genes encoding MBW complex in response to light

**Figure 4:**
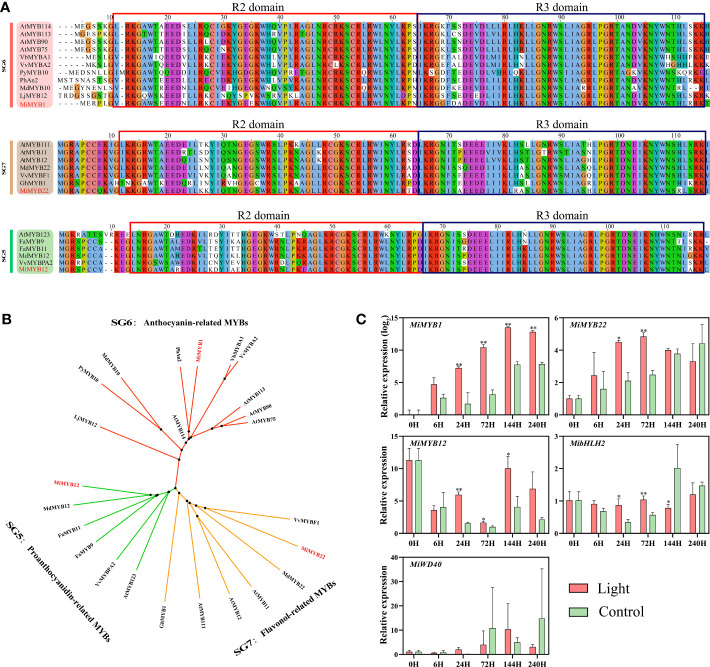
**(A)** Multiple sequence alignment of flavonoids-related MYBs in mango and other plant species. The R2R3 domain is boxed. The mango proteins are highlighted in red. **(B)** Phylogenetic tree derived from amino acid sequences of flavonoids-related MYBs in mango and other plant species. The mango proteins are highlighted in red. All sequences were retrieved from NCBI database with accessions as follows: SG6: *Arabidopsis thaliana* AtMYB114 (Q9FNV8.1), AtMYB113 (Q9FNV9.1), AtMYB90 (Q9ZTC3.1) and AtMYB75 (Q9FE25.1); *Vitis betulifolia* VbMYBA1 (AGH68552.1); *Vitis vinifera* VvMYBA2 (BAD18978.1); *Pyrus pyrifolia* PyMYB10 (ALN66630); *Petunia x hybrida* PhAN2 (AAF66727); *Malus domestica* MdMYB10 (ACQ45201.1); *Lilium japonicum* var. *Abeanum* LjMYB12 (BAP00661.1). SG7: *Arabidopsis thaliana* AtMYB111 (NP_199744.1), AtMYB11 (Q9LZK4.1) and AtMYB12 (NP_182268.1); *Malus domestica* MdMYB22 (AAZ20438.1), *Vitis vinifera* VvBF1 (NP_001267930.1); *Gossypium hirsutum* GhMYB1 (NP_001313761.1). SG5: *Arabidopsis thaliana* AtMYB123 (Q9FJA2.1); *Fragaria x ananassa* FaMYB9 (AFL02460.1), FaMYB11 (AFL02461.1); *Malus domestica* MdMYB12 (XP_008337875.1); *Vitis vinifera* VvMYBPA2 (NP_001267953.1). **(C)** Effect of light treatment on the expression of flavonoids-related *MiMYBs*, *MibHLH2*, and *MiWD40* in ‘Zill’ mango peel. Each value represents the mean ± standard deviation of three biological replicates. * indicates significant difference (*p*-value < 0.05), and ** indicates very significant difference (*p*-value < 0.01) between control and treatment, as determined by Student’s *t*-test.

The MYB regulating anthocyanins biosynthesis in mango has already been reported by Kanzaki et al. (2020), named MiMYB1, while the flavonols and proanthocyanidins-related MYBs are still unknown. In this study, we used MdMYB12 and MdMYB22, which have been proven to regulate proanthocyanidins and flavonols biosynthesis in apple by Wang et al. (2017), to search for the mango homologs MYBs in the mango genome database (Wang et al., 2020a). Finally, Mi12g07270.1, and Mi09g04990.1 were defined as the homologs of apple MdMYB12 and MdMYB22 in mango, named MiMYB12 and MiMYB22, respectively. Multiple sequence alignment showed that MiMYB1, MiMYB22, and MiMYB12 shared a very conserved R2R3 domain with the relevant MYBs in other plant species including Arabidopsis, strawberry, grape, and apple (**Figure 4A**). Phylogenetic analysis based on the full protein sequence showed that MYBs from Subgroups (SGs) 5, 6, and 7 were clustered together, and mango MiMYB1, MiMYB12 and MiMYB22 showed the closest relationship with the homologs from petunia, apple and grape, respectively (**Figure 4B**).

The expression of *MiMYB1*, *MiMYB12*, and *MiMYB22* was all induced by light, with the most significant response by *MiMYB1* (**Figure 4C**). *MiMYB1* transcription in light-treated samples was up-regulated from day 1, and lasted during the whole experiment (**Figure 4C**). The increased expression of *MiMYB12* and *MiMYB22* by light treatment was detected at day 1, day 3, and day 6, and day 1 and day 3, respectively (**Figure 4C**). *MibHLH2* expression was slightly increased at day 1 and day 3, and subsequently slightly decreased at day 6, while the expression of *MiWD40* showed no response to light (**Figure 4C**).

### Identification of mango HY5 and HYH, and expression of light signal genes in response to light

**Figure 5:**
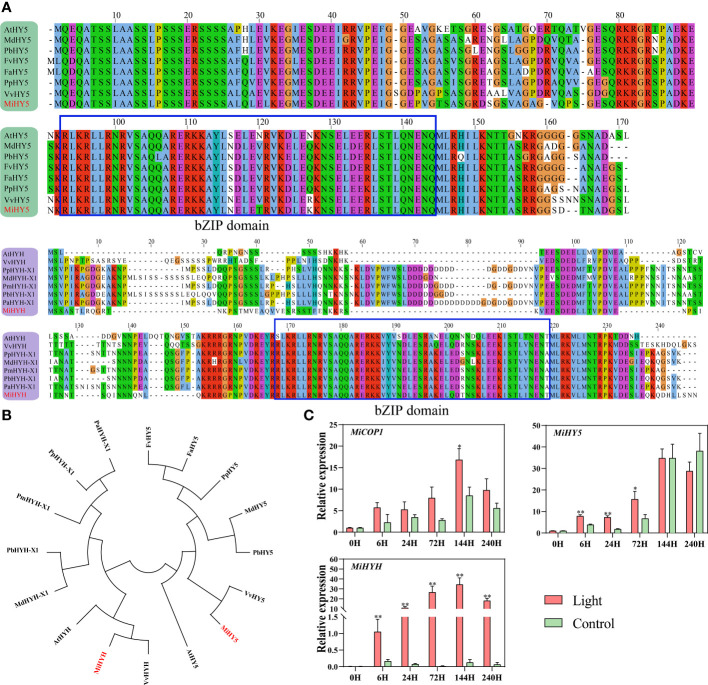
**(A)** Multiple sequence alignment of HY5 and HYH proteins in mango and other plant species. The bZIP domain is boxed. The mango proteins are highlighted in red. **(B)** Phylogenetic tree derived from amino acid sequences of HY5 and HYH in mango and other plant species. The mango proteins are highlighted in red. All sequences were retrieved from NCBI database with accessions as follows: *Arabidopsis thaliana* AtHY5 (O24646.1) and AtHYH (OAP05786.1); *Malus domestica* MdHY5 (NP_001280752.1) and MdHYH-X1 (XP_008369576.1); *Pyrus x bretschneideri* PbHY5 (XP_009355719.1) and PbHYH-X1 (XP_009353456.1); *Fragaria vesca subsp. Vesca* FvHY5 (XP_004291469.1); *Fragaria x ananassa* FaHY5 (AKG58815.1); *Prunus persica* PpHY5 (XP_020411091.1) and PpHYH-X1 (XP_020409867.1); *Vitis vinifera* VvHY5 (XP_010648648.1) and VvHYH (AHX24181.1); *Prunus mume* PmHYH-X1 (XP_008222315.1); *Prunus avium* PaHYH-X1 (XP_021812250.1). **(C)** Effect of light treatment on the expression of flavonoids-related *MiCOP1*, *MiHY5*, and *MiHYH* in ‘Zill’ mango peel. Each value represents the mean ± standard deviation of three biological replicates. * indicates significant difference (*p*-value < 0.05), and ** indicates very significant difference (*p*-value < 0.01) between control and treatment, as determined by Student’s *t*-test.

Using the apple MdHY5 (NP_001280752.1) and MdHYH (XP_008369576.1) sequences, the respective homologs in mango were identified, named MiHY5 (mango009397) and MiHYH (mango023606). Multiple sequence alignment showed that both MiHY5 and MiHYH contain a conserved bZIP domain (**Figure 5A**). The phylogenetic tree showed that HY5 and HYH were clustered into two groups, and all the HY5 or HYH proteins were clustered together (**Figure 5B**). Q-PCR analysis showed that *MiCOP1* expression was only induced by light at 6 hours of treatment (**Figure 5C**). The transcription of *MiHY5* was mainly up-regulated by light at the early stages of treatment, i.e. from 6 hours to 3 days, while *MiHYH* expression was significantly in response to light during the whole treatment (**Figure 5C**).

### Correlation analysis between flavonoids contents and gene expression

**Figure 6:**
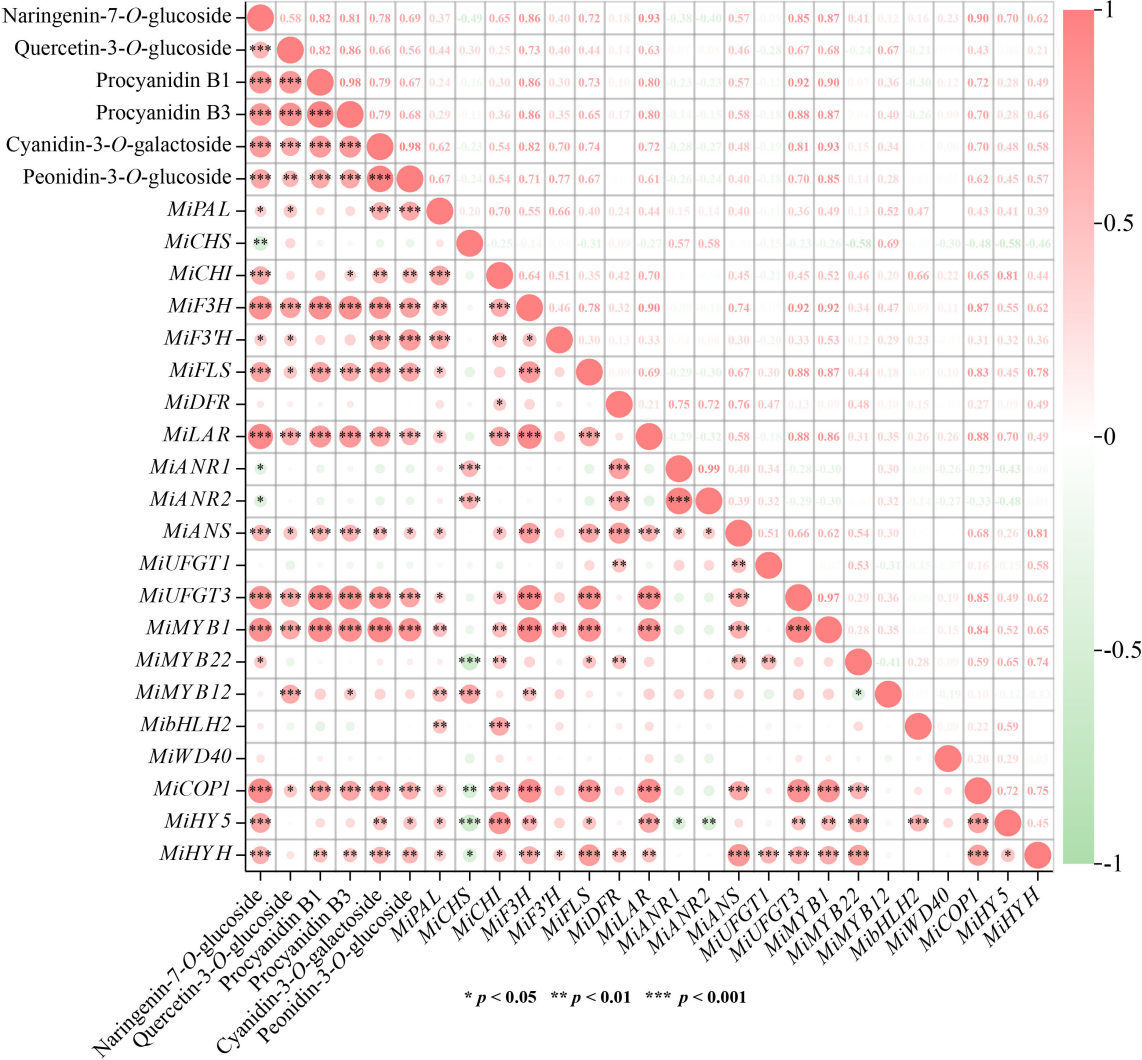
Correlation analysis among flavonoids contents and gene expression. Positive and negative correlations are indicated by colors, with red representing positive and green representing negative (*, *p* < 0.05, **, *p* < 0.01 and ***, *p* < 0.001). The numbers represent the Pearson correlation coefficient between two variables.

Correlation analysis was carried out to understand the relationship among flavonoids contents and gene expression. Results showed that 6 flavonoids components exhibited high positive correlation with each other (*r* > 0.5, *p* < 0.01) (**Figure 6**). The most significant correlations were observed between two proanthocyanidins, i.e. procyanidin B1 and procyanidin B3 (*r* = 0.98, *p* < 0.001), and two anthocyanins, i.e. cyanidin-3-*O*-galactoside, and peonidin-3-*O*-glucoside (*r* = 0.98, *p* < 0.001), respectively (**Figure 6**). For correlations between flavonoids contents and gene expression, *MiF3H*, *MiLAR*, *MiUFGT3*, and *MiMYB1* were highly positively correlated with all the flavonoids components (*r* > 0.6, *p* < 0.001) (**Figure 6**). *MiFLS* was highly correlated to naringenin-7-*O*-glucoside concentration (*r* = 0.72, *p* < 0.001), but lowly correlated to quercetin-3-*O*-glucoside concentration (*r* = 0.44, *p* < 0.05) (**Figure 6**). Procyanidin B1 and procyanidin B3 showed high correlation with *MiLAR*, but low correlation with *MiANR1* and *MiANR2* (**Figure 6**). Cyanidin-3-*O*-galactoside, and peonidin-3-*O*-glucoside showed high correlation with *MiUFGT3*, but low correlation with *MiUFGT1* (**Figure 6**). *MiMYB12* was highly correlated with quercetin-3-*O*-glucoside (*r* = 0.67, *p* < 0.001), while *MiMYB22* showed low correlation with flavonoids concentration (**Figure 6**).

## Discussion

### Light induces the flavonoids accumulation in mango fruit peel but also indicates the competition among different flavonoids compositions

Light is one of the most crucial environmental factors not only regulates plant growth and development, but also induces secondary metabolites accumulation, for instance, flavonoids. Numerous preharvest and postharvest studies have shown that light promotes the total flavonoids concentration in plant (Huang et al., 2009; Qian et al., 2021), as well as different flavonoids components, including flavonols, proanthocyanidins and anthocyanins (Qian et al., 2013; Scattino et al., 2014; Shi et al., 2021). In this study, light induced the accumulation of flavonols, proanthocyanidins and anthocyanins in mango peel (**Figure 2**), which was similar with the previous studies. In addition, the accumulation of flavonols and proanthocyanidins peaked at day 6 and subsequently decreased towards day 10 (**Figure 2**), while the obvious red coloration and anthocyanins accumulation started at day 6, continuously increased, and peaked at day 10 (**Figure 2**), indicating the competition among different flavonoids compositions. Flavonols, proanthocyanidins and anthocyanins are synthesized from different branches which are derived from the same pathway, and after accumulating certain amount of flavonoids, plants fine-tune the pathway to fulfill the demand of growth and development instead of over-accumulating flavonoids. For example, many fruits accumulate flavonols and proanthocyanidins as the main flavonoids components at the early developmental stage to provide astringent taste against early feeding (Jaakola, 2013), while anthocyanins become the dominant composition in ripe fruits to attract insects and animals for seed dispersal (Harborne and Williams, 2000). The competition between proanthocyanidins and anthocyanins is more obvious because they are derived from the same precursor, anthocyanidins. Therefore, the increased content of proanthocyanidins or anthocyanins usually leads to the decrease of the other substance (Xie et al., 2003; Han et al., 2012).

### Flavonoids accumulation is associated with the up-regulation of the key structural genes

Among all the EBGs, *MiF3H* was continuously induced by light (**Figure 3A**), ensuring the sufficient precursor accumulation for flavonoids biosynthesis, i.e. dihydroflavonols. Dihydroflavonols are important intermediate products in flavonoids pathway, which can be directly catalyzed by FLS to form flavonols or further processed for proanthocyanidins and anthocyanins biosynthesis. *MiFLS* expression was also tremendously induced by light resulting in the increased accumulation of flavonols (**Figures 2**, **3B**). It has been showed that antisense expression of a *FLS* gene in petunia could inhibit flavonols accumulation and switched the petal color from light pink to red (Holton et al., 1993), while over-expression of a *Camellia nitidissima FLS* gene in tobacco promoted flavonols accumulation and change floral color from pink to white or light yellow (Zhou et al., 2013). The enhanced expression of *MiANS* in light-treated fruit peels was detected from 6 hours to 10 days (**Figure 3B**). ANS is crucial for both proanthocyanidins and anthocyanins biosynthesis since it catalyzes the formation of anthocyanidins, which can be further catalyzed by ANR to form proanthocyanidins, or by UFGT to form anthocyanins. The up-regulation of *MiUFGT1* and *MiUFGT3* mainly occurred form day 3 to day10 (**Figure 3B**), while *MiANR1* and *MiANR2* responded to light at the early stage (**Figure 3B**), indicating most anthocyanidins were converted to anthocyanins instead of proanthocyanidins at the late stage of light treatment, which was correlated to the increasing accumulation of anthocyanins from day 6 to day 10 (**Figure 2**). *MiLAR* expression was highly correlated with proanthocyanidins concentration (**Figures 2**, **3B**, **6**), indicating the conversion from leucoanthocyanins to proanthocyanidins catalyzed by LAR, rather than from anthocyanidins by ANR, was predominant during proanthocyanidins biosynthesis. It has been reported that overexpression of an apple *ANR* gene in tobacco suppressed the tobacco *LAR* expression, which also indicated the competition between these two NAPDH-dependent reductases (Han et al., 2012).

### MYBs play essential roles in light-induced flavonoids biosynthesis

By regulating the expression of flavonoids biosynthetic genes, MYBs are regarded as the most important TFs mediating flavonoids synthesis, and this process is often in response to light signal. Over-expression of a light-induced Tartary buckwheat *FtMYB6* gene in Tartary buckwheat hairy roots and tobacco could significantly increase the accumulation of flavonols (Yao et al., 2020). Over-expression of Arabidopsis *AtMYB111* in tobacco promoted flavonols accumulation, which requires light (Pandey et al., 2014). UV-B light responsive *MYB134* promotes proanthocyanidins synthesis in poplar by binding to the promoter regions of PA pathway genes including *PAL* and *ANR* (Mellway et al., 2009). Compared with flavonols and proanthocyanidins, anthocyanins are more widely studied due to their great contribution to the plants coloration, and MYBs regulating light-induced anthocyanins biosynthesis has been reported in diverse fruit species including apple (Li et al., 2012; Bai et al., 2016), pear (Feng et al., 2010; Ni et al., 2019), blood orange (Huang et al., 2019), Chinese bayberry (Niu et al., 2010), litchi (Lai et al., 2014), and mango (Kanzaki et al., 2020). In this study, the putative MYBs regulating flavonols and proanthocyanidins biosynthesis, i.e. MiMYB22 and MiMYB12, have been identified, respectively (**Figures 4A, B**), and the expression of *MiMYB1*, *MiMYB22* and *MiMYB12* was significantly increased by light treatment (**Figure 4C**), suggesting these MYBs probably regulate light-induced flavonoids biosynthesis.

### COP1, HY5 and HYH are key upstream regulators of light-induced flavonoids biosynthesis

After being sensed by different photoreceptors, light signal pathway is transduced by COP1, HY5, and HYH. Under darkness, COP1 is located in the nucleus to degrade flavonoids-related regulators such as HY5 and MYBs *via* ubiquitination (Osterlund et al., 2000; Saijo et al., 2003; Li et al., 2012; Maier et al., 2013). Under light conditions, COP1 is translocated to cytoplasm leading to the accumulation of TFs such as HY5 and MYBs and subsequent flavonoids biosynthesis (Jiao et al., 2007; Li et al., 2012). In this study, the expression of *MiCOP1* was induced by light at day 6, but showed no difference between light-treated sample and control sample at the other time points (**Figure 5C**), indicating that COP1 functions mainly through the protein subcellular localization instead of gene expression.

HY5 and HYH have also been widely reported to regulate light-induced flavonoids accumulation. In apple, *MdHY5* regulates light-induced anthocyanins biosynthesis *via* binding to E-box and G-box motifs in the promoter region of *MdMYB10* (An et al., 2017). PyHY5 is involved in the light-induced anthocyanins biosynthesis in pear by promoting the expression of *PyWD40* and *PyMYB10* (Wang et al., 2020b). As the homolog of HY5, HYH has also been shown to contribute to the light-induced flavonoids biosynthesis in peach (Zhao et al., 2022) and Arabidopsis (Holm et al., 2002). In this study, both *MiHY5* and *MiHYH* responded very quickly to light and the increased expression lasted for quite a long time (**Figure 5C**), indicating the essential roles of HY5 and HYH during the light signal transduction.

## Conclusions

Postharvest light treatment promoted the red peel coloration and increased the total soluble solids content and firmness in ‘Zill’ mango fruit. Metabolites profile showed that the accumulation of flavonols, proanthocyanidins and anthocyanins was also induced by light, as well as the key flavonoids biosynthetic genes including *MiF3H*, *MiFLS*, *MiLAR*, *MiANS*, *MiUFGT1*, and *MiUFGT3*. The flavonoids related MYBs, and essential light signal TFs HY5 and HYH, were also identified in mango, and their expression showed a light responsive pattern. Our results provide some molecular clues about light-induced flavonoids accumulation in mango.

## Data availability statement

The original contributions presented in the study are included in the article/**Supplementary Material**, further inquiries can be directed to the corresponding author/s.

## Author contributions

Conceptualization: WZ, HW, and MQ. Methodology: WZ, HW, CY, BS, BZ, XM, and KZ. Data curation: WZ, HW, CY, BS, BZ, XM, and KZ. Writing—original draft preparation: WZ, HW, CY, BS, BZ, XM, KZ, and MQ. Writing—review and editing: WZ, HW, CY, BS, BZ, XM, KZ, and MQ. Funding acquisition: HW and MQ. All authors contributed to the article and approved the submitted version.
